# Impact of Fatty Acids on Obesity-Associated Diseases and Radical Weight Reduction

**DOI:** 10.1007/s11695-021-05789-w

**Published:** 2021-11-23

**Authors:** Małgorzata Wrzosek, Zuzanna Zawadzka, Ada Sawicka, Barbara Bobrowska-Korczak, Agnieszka Białek

**Affiliations:** 1 Department of Biochemistry and Pharmacogenomics, Faculty of Pharmacy, Medical University of Warsaw, 1 Banacha Street, 02-097 Warsaw, Poland; 2 Centre for Preclinical Research, Medical University of Warsaw, 1 Banacha Street, 02-097 Warsaw, Poland; 3 Department of Family Medicine, Internal Medicine and Metabolic Bone Diseases, Center of Postgraduate Medical Education, 00-416, Warsaw, Poland; 4 Department of Bromatology, Faculty of Pharmacy, Medical University of Warsaw, 1 Banacha St., 02-097 Warsaw, Poland; 5 Institute of Genetics and Animal Biotechnology PAS, Postepu 36A, Jastrzębiec, 05-552 Magdalenka, Poland

**Keywords:** Fatty acids, Bariatric surgery, Obesity, Metabolic disturbances

## Abstract

**Purpose:**

Fatty acids (FA), particularly polyunsaturated (PUFA) ones, are involved in the regulation of glycemic control, lipid metabolism, and inflammation. The aim of the study was to assess patient FA profile in relation to obesity, lipid and carbohydrate metabolism disturbances, and weight loss.

**Materials and Methods:**

The studied group consisted of 51 patients with extreme obesity, 23 of whom achieved radical weight reduction within 1 year after a laparoscopic sleeve gastrectomy (LSG). FA levels were determined using gas chromatography with flame ionization detection.

**Results:**

Patients with extreme obesity and higher serum PUFA content have lower serum levels of SFA and MUFA (especially myristic, palmitic, lignoceric acids and palmitoleic, oleic acids), as well as lower triglyceride and higher HDL-cholesterol concentrations and it was not influenced by CEPT Taq1B variant. At baseline, the fatty acid profile of patients with type II diabetes differ from patients with dyslipidemia. In patients who had lost weight, significantly lower levels of selected saturated FA and major trans-fatty acid, elaidic, were found. Moreover, the proportion of PUFA was increased.

**Conclusion:**

In extreme obesity, higher PUFA exert their favorable effects on serum lipids. Significant weight reduction after the bariatric surgery is associated with beneficial changes in the fatty acid profile.

**Graphical Abstract:**

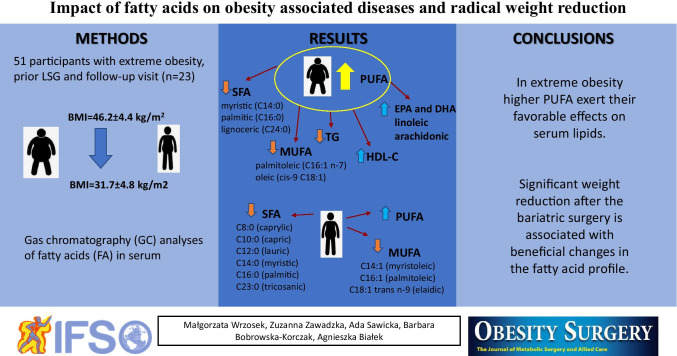

## Introduction

According to the World Health Organization, the number of people with obesity throughout the world has almost tripled since 1975 [1]. Obesity was once considered a problem of rich and intensively developing countries, but it is now thought to affect people with low and medium socioeconomic status [2]. In individuals with obesity, the amount of energy supplied is much higher than the body's need, which results in excessive fat accumulation due to hypertrophy and/or adipocyte hyperplasia [3]. An energy imbalance results from taking in more calories than is required for body health and function. Every year, the percentage of overweight people increases due to the popularity of consuming foods high in fat and carbohydrates. Undoubtedly, body weight is also significantly influenced by eating habits, e.g., overeating, too long or too short breaks between meals, and eating to relieve negative emotions or anxiety [4]. In addition, people who have limited physical activity tend to gain weight. Their volume of lipid droplets in skeletal muscle is increased. Lower rates of triglyceride turnover, diminished oxidative enzyme activity, and lipid oxidation are also observed. These mechanisms lead to adipose tissue retention [5, 6]. The energy contained in dietary fat comes mainly from saturated (SFA), monounsaturated (MUFA), and polyunsaturated (PUFA) fatty acids [7]. The most common SFA are palmitic and stearic acid, which are usually consumed in the diet in excessive amounts [8]. A recommended intake for these acids has not yet been established, nor has an upper safe dietary level. SFA are considered to adversely affect human health, because any increase in their content carries the risk of coronary heart disease or an increased concentration of low-density lipoprotein (LDL) cholesterol [9, 10]. Fatty acids are stored in lipid droplets within adipocytes in the form of triglycerides. Excess fatty acid accumulation leads to an increase in mature adipocyte size [11]. Hypertrophic adipocytes tend to be insulin resistant. This results in greater lipolysis with less lipogenesis. Thus, increased fatty acid flux away from adipose tissue represents a key feature in the pathophysiology of the metabolic complications in obese individuals, such as poor glycemic control, dyslipidemia, and inflammation. MUFAs coexist with SFA in foods, especially in animal products. The most important food sources of MUFA are olive oil, animal fats (oleic acid, myristoleic, and palmitoleic acids) and rapeseed oil (erucic acid). PUFA constitute an important group of human nutrients. Unlike SFA, not all PUFA can be synthesized by human and animal tissues. This is due to a lack of special enzyme systems (Δ^12^-desaturase and Δ^15^-desaturase) which would insert unsaturated bonds in positions 3 and 6 of the carbon chain. In animal tissues, it is only possible to remodel such chains and produce other polyunsaturated acid chains from them. Therefore, linoleic acid (LA) and α-linolenic acid (ALA) belong to the so-called EFAs (essential unsaturated fatty acids) and they must be derived from dietary sources [12, 13]. Unsaturated fatty acids are irreplaceable in young organisms for proper growth and development processes and contribute to the maintenance of good health throughout life. They have a role in building cellular membranes throughout the body and stimulate multiple signaling events in various tissues, mainly via eicosanoids derived from them and via direct effects on gene expression [14]. Moreover, studies have shown anti-inflammatory properties for long-chain n-3 polyunsaturated fatty acids (n-3 PUFA) [15, 16], which directly modulate the activity of the key pro-inflammatory transcription factor NF-κB [17]. EPA and DHA have established cardiovascular benefits [18–20]. Fatty acids in serum cholesteryl esters regulate cholesterol homeostasis by participating in the transport and oxidation of cholesterol [21]. For example, n-3 PUFA reduce endogenous triglyceride synthesis and enhance the blood clearance of triglyceride-rich particles, stimulate the inhibition of lipogenesis, promote fat oxidation and reduce fat deposition, thereby counteracting obesity and dyslipidemia [22, 23]. Omega‑3 fatty acids may also reduce insulin resistance, which is of importance in diabetes prevention [24, 25]. Thus, the aim of the study was to assess fatty acid profile in relation to obesity, obesity-associated diseases, and weight reduction. We considered that an investigation in obese individuals might be important in determining an individual’s risk of developing lipid and carbohydrate disturbances and might also provide useful information about the role of selected fatty acids in metabolic alterations, inflammation, and maintaining weight loss. We verified also the influence of cholesteryl ester transfer protein (CETP) gene variant (Taq1B) on fatty acid contents. It is speculated that variations in the CETP gene may modulate the effects of dietary components on metabolic traits and individuals homozygous for the B2 genotype have higher HDL-C concentrations and reduced risk of the metabolic syndrome than those with the B1 homozygous genotype [26, 27]. In the present study, chemometric approach has been proposed as an objective method for the evaluation and data interpretation of large data sets of fatty acid share in serum samples of three groups of patients (individuals with obesity, with obesity and co-existing dyslipidemia, and with obesity and co-existing diabetes).

## Materials and Methods

### Study Group and Study Design

Unrelated subjects with morbid obesity (BMI = 46.2 ± 4.44; range 40.0–56.0), over 35 years of age, were consecutively recruited from patients prior to bariatric surgery. A detailed history of obesity and a full physical examination was obtained for each patient. Obesity was classified according to the World Health Organization criteria [28]. In all subjects, anthropometric measurements (body weight and height) were taken and body mass index (BMI) was calculated as the ratio of weight (kilograms) to the square of height (meters). The data on DXA-derived measures of total body fat (fat mass expressed as % fat mass and kg) was available for the whole group of participants. Within the 12 months following a laparoscopic sleeve gastrectomy (LSG), only those patients with marked weight loss were enrolled in prospective, observational study. The percent changes of BMI values were determined by the Eq. 100 × (BMI at baseline – BMI at follow-up)/BMI at baseline. The study was carried out in accordance with the principles of the Declaration of Helsinki. The whole study protocol was approved by the Institutional Bioethics Committees. Subjects' weight and height were measured, and body mass index (BMI) was calculated at pre‐surgery and follow‐up visits. Determination of dyslipidemia was based on a current or previous medical diagnosis according to the National Cholesterol Education Program-Adult Treatment Panel III [29]. Patients were classified as diabetics based on the review of medical records (an average fasting plasma glucose concentration ≥ 126 mg/dl on two occasions [30], previous diagnosis of diabetes by a physician, and current use of diabetes medications) and confirmed by current medical examination. Only patients whose type II diabetes was diagnosed after the age of 30 were included in the analyses. Criteria for exclusion from the study were as follows: acute endocrine dysfunction, pregnancy, previous bariatric surgery, and alcoholism. In addition, individuals with prediabetes were excluded from the study [30]. The primary outcome was the relation of FA to obesity-associated disturbances of glucose and lipid metabolisms and to changes in serum FA after significant weight reduction.

### Biochemical Analysis

Blood samples were drawn in the morning after 12-h overnight fasting at the time of surgery (pre‐surgery) and during the follow‐up visit. Biochemical parameters measured included total cholesterol (TC), HDL-cholesterol (HDL-C), LDL-cholesterol (LDL-C), triglycerides (TG), fasting blood glucose (FBG), HbA1c (%), folic acid, vitamin B12, C-reactive protein (CRP), aspartate aminotransferase (ASP), alanine aminotransferase (ALT), insulin, and IL-6. Laboratory analyses were performed by routine laboratory methods. Serum levels of interleukin 6 (IL-6) was determined by the ELISA method using the Diaclone Human IL-6 High Sensitivity ELISA kit (950.035.192). Serum levels of insulin were determined by the ELISA method using the DRG® Insulin ELISA (EIA-2935) kits. Insulin resistance was assessed using the homeostasis model assessment [HOMA-IR index = (fasting glucose in mmol/L * fasting insulin in µIU/mL)/22.5] [31].

### Fatty Acid Analysis in Serum

Fatty acid analyses were performed with gas chromatography (GC) using a gas chromatograph (GC-17A Shimadzu, Japan) equipped with a capillary column (BPX 70; 60 m × 0.25 mm i.d., film thickness 0.20 μm, SGE, Ringwood, Australia) and a flame-ionization detection (FID). Helium (Multax) was the carrier gas. The initial oven temperature was 140 °C for 1 min after that increased by 20 °C/min to 200 °C and held for 20 min and increased by 5 °C/min to 220 °C held for 25 min. The injector was heated to 250 °C, and the detector to 270 °C. Fatty acid methyl esters (FAME) standards (Supelco 37 Component FAME Mix, Sigma, St. Louis, MO, USA), CLA FAME reference standard (Nu-Chek-Prep, INC., USA), PA methyl ester reference standard (methyl punicate, Matreya LLC, USA) were used to identify the fatty acids present in samples. The serums were thawed only once, and samples of 100 μl were trans-esterificated according to the procedure of Bondia-Pons et al. [32]. with minor modifications. Without prior lipid extraction, the serum samples were hydrolyzed by heating with 2.5 ml of sodium methoxide in methanol (0.5 mol/l) at 80 °C for 10 min. FA were converted into methyl esters by heating with 2.5 ml of 14% boron trifluoride-methanol reagent at 80 °C for 3 min. FAME were isolated with hexane (2 × 0.5 ml) after adding 1.0 ml of saturated sodium chloride solution. Organic extracts were dried over anhydrous sodium sulfate and evaporated to dryness under a stream of nitrogen. FAME were diluted in 20 μl of hexane and stored at − 20 °C until being analyzed. Results were expressed as percentage of total fatty acids present in serum. The fatty acids determined in patients are presented in Table 2.

### CETP Genotyping

DNA was extracted from the leukocytes of the whole blood leucocytes with a DNA extraction kit (A&A Biotechnology, Poland). The Taq1B genotype (rs708272) of the cholesteryl ester transfer protein (CETP) gene was determined by a polymerase chain reaction using primers as described by Mohrschlad et al. [33]. DNA was amplified in a 25-µl reaction mixture containing 25 pmol of each primer, 100 ng genomic DNA, 0.2 mmol/l of each dNTP, 1.5 mM MgCl2, and 0.2 U Taq polymerase (BioTaq, Bioline Reagents, UK). The amplification was performed at 94 °C for 4 min, followed by 35 cycles at 94 °C for 30 s, 64 °C for 30 s, and 72 °C for 1.5 min. A fragment of 505 bp in intron 1 of the CETP gene was amplified by polymerase chain reaction (PCR). The PCR products were subjected to restriction enzyme analysis by digestion with Taq1 restriction endonucleases (Fermentase Canada) at 65 °C for 1 h. The fragments were separated by electrophoresis on a 2.5% agarose gel and stained with ethidium bromide. DNA fragments were visualized by UV illumination. The resulting fragments were 505 bp and 90 bp for the “B1’ allele and 505 bp for the uncut “B2” allele.

### Statistical Analysis

Statistica 13.0 (StatSoft, Poland) was used to conduct a statistical analysis of the results. The normality assumptions were estimated with Shapiro–Wilk’s test, and whenever the normality and variance homogeneity assumptions were fulfilled, one-way analysis of variance (ANOVA) with post hoc Tukey’s test was used to determine the relationships between the 3 groups. If the assumptions of the analysis of variance were not met, the non-parametric Kruskal–Wallis test, which is a non-parametric equivalent of one-way ANOVA, with post hoc multiple comparison test was used. Pearson’s χ2 tests were used to find a link between the CEPT Taq1B variant and PUFA level in obese subjects. Paired *t*-test was used to compare values at baseline with 12 months post‐surgery values. Subjects were divided into quintiles of PUFA levels; Mann–Whitney rank tests and *t*-tests were used to assess differences between the groups. Spearman’s coefficients were used to estimate potential correlations between CRP (C-reactive protein) and FFA contents and selected biochemical parameters. In order to better understand the data trends, fatty acid profiles were considered as chemical descriptors to study a possible discrimination of serum samples from patients with obesity, with obesity and dyslipidemia, and with obesity and type II diabetes. Prior to chemometric analyses, the original data were transformed into natural logarithms and then standardized. Similarity analysis was performed for variables differing significantly among those three groups of patients, and grouping of features and objects was carried out to prepare heat map. Moreover, in order to obtain appropriate classification rules for serum samples into obesity, obesity with dyslipidemia, and obesity with type II diabetes, a linear discriminant analysis (LDA) was performed. Relevant discriminant functions were calculated in a stepwise progressive method, with the adopted tolerance value 1- *R*^2^ = 0.01 to optimize LDA.

## Results

Biochemical characteristics and anthropometric parameters of 51 participants with extreme obesity and prior bariatric surgery are presented in Table 1. The mean BMI value of the subjects was 46.20 ± 4.44 kg/m^2^. The study group consisted of 37 women and 14 men with the mean age 44.8 ± 6.6 years. In our experiment, we analyzed 36 fatty acids, including 15 saturated fatty acids (SFA), 9 monounsaturated fatty acids (MUFA), and 12 polyunsaturated fatty acids (PUFA). Participants were divided into quartiles of PUFA level (Table 1). It was shown that obese patients in the highest quartile of serum PUFA (% PUFA > 32.84%) had a significantly higher HDL-cholesterol levels (*p* = 0.01) and lower triglyceride concentration (*p* = 0.03), even though they had higher BMIs. Genotype B2B2 of CEPT Taq1B variant was not associated with higher PUFA level (Table 1).

**Table 1:** Basic characteristic of patients (*n* = 51) and differences between these parameters in patients with high (> Q3) and low (≤ Q3) PUFA levels

Parameters	Total (*n* = 51)	PUFA ≤ 32.84% (Q3) (*n* = 39)	PUFA > 32.84% (Q3) (*n* = 12)	*p value*
CEPT Taq1B variant (number of B2B2 homozygous patients)	n = 10 (19.6%)	n = 7 (18%))	n = 3 (25%)	0.37
Weight (kg)	127.70 ± 18.52	125.39 ± 17.81	127.20 ± 9.76	0.27
BMI (kg/m^2^)	46.20 ± 4.46	**45.28 ± 4.28**	**49.52 ± 3.62**	**0.04**
Fat mass (kg)	58.69 ± 9.33	57.9 ± 9.82	61.65 ± 6.97	0.35
Fat (%)	47.40 ± 4.36	46.41 ± 3.44	48.39 ± 2.87	0.29
Lean mass (kg)	62.66 ± 9.25	63.47 ± 11.00	62.88 ± 9.254	0.89
HbA_1c_ (%)	5.84 ± 0.70	5.89 ± 0.72	5.65 ± 0.66	0.30
Fasting glucose (mg/dl)	97.20 ± 19.14	98.92 ± 20.55	91.70 ± 13.01	0.26
Insulin (µIU/ml)	25.74 ± 17.48	26.51 ± 18.56	21.61 ± 9.24	0.50
HOMA-IR	6.26 ± 5.07	6.58 ± 5.41	4.52 ± 1.86	0.30
ESR (mm/h)	16.59 ± 8.9	15.36 ± 7.92	19.67 ± 11.52	0.24
CRP (mg/dl)	10.59 ± 6.6	10.02 ± 6.57	12.02 ± 6.69	0.36
IL6 (pg/ml)	4.01 ± 3.20	3.76 ± 2.72	4.07 ± 3.40	0.23
25(OH)D (ng/ml)	16.75 ± 11.68	16.58 ± 7.20	17.31 ± 20.98	0.85
AspAt (U/l)	29.14 ± 10.13	30.17 ± 10.74	27.83 ± 8.19	0.24
AlAt (U/l)	43.86 ± 15.17	44.70 ± 16.23	41.36 ± 11.77	0.29
Total cholesterol (mg/dl)	180.59 ± 41.61	180.51 ± 39.06	180.83 ± 51.00	0.98
HDL-cholesterol (mg/dl)	42.09 ± 10.42	**40.13 ± 9.44**	**48.49 ± 11.28**	**0.01**
LDL-cholesterol (mg/dl)	112.55 ± 36.26	112.62 ± 35.01	112.33 ± 41.71	0.98
Triglycerides (mg/dl)	132.63 ± 58.65	**142.54 ± 62.91**	**100.42 ± 22.02**	**0.03**
Folic acid (ng/ml)	7.59 ± 3.54	8.11 ± 3.72	5.93 ± 2.31	0.06
B_12_ (pg/mL)	319.04 ± 111.70	321.21 ± 116.19	312.00 ± 100.06	0.81
Systolic b. p. (mmHg)	139.88 ± 20.87	136.11 ± 20.96	140.50 ± 22.72	0.22
Diastolic b. p. (mmHg)	79.28 ± 9.53	77.03 ± 9.44	75.50 ± 9.21	0.74

The values are given as the means ± standard deviations (SD). Bold data indicate significance difference. Abbreviations: *b.p.* blood pressure, *BMI* body mass index, *HbA1c* glycosylated hemoglobin, *HDL-cholesterol* high-density lipoprotein cholesterol, *LDL-cholesterol* low-density lipoprotein cholesterol, *ESR* erythrocyte sedimentation rate, *CRP* C-reactive protein, *AspAt* aspartate aminotransferase, *AlAt* alanine aminotransferase.

Mann–Whitney rank tests and *t*-tests were used where appropriate. Pearson’s χ2 tests were used to find a link between CEPT Taq1B B2B2 genotype and PUFA level. *P* values were considered significant when *p* < 0.05.

Palmitic (C16:0), oleic (C18:1 n-9), LA (linoleic, C18:2 n-6), and stearic (C18:0) acids were found to be the main fatty acids in the serum of the patients in the study (Table 2). In addition, patients with higher total percent PUFA had a significantly lower percentage of SFA, especially myristic (C14:0), palmitic (C16:0), lignoceric (C24:0), and lower percentages of MUFA, i.e., palmitoleic (C16:1 n-7) and oleic acids (cis-9 C18:1), whereas they had higher percentages of linoleic (LA, C18:2 n-6), arachidonic (C20:4 n-6), EPA (eicosapentaenoic), and DHA (docosahexaenoic) compared to patients with lower PUFA levels (% PUFA ≤ Q3). We observed that total content of SFA and MUFA was lower in patients with a higher percent of PUFA and they had higher serum total n-3 as well as n-6 PUFA concentrations (Table 2).

**Table 2:** Fatty acid composition in serum of patients (*n* = 51) and differences between these parameters in patients with high (> Q3) and low (≤ Q3) PUFA levels

Fatty acids (%)	Total (*n* = 51)	PUFA ≤ 32.84% (Q3) (*n* = 39)	PUFA > 32.84% (Q3) (*n* = 12)	*p value*
SFA				
C8:0 (caprylic)	1.32 ± 0.59	1.31 ± 0.62	1.36 ± 0.52	0.79
C10:0 (capric)	1.62 ± 0.55	1.64 ± 0.60	1.58 ± 0.35	0.76
C12:0 (lauric)	0.90 ± 0.31	0.91 ± 0.35	0.87 ± 0.17	0.70
C13:0 (tridecylic)	0.15 ± 0.20	0.18 ± 0.22	0.11 ± 0.16	0.62
C14:0 (myristic)	0.80 ± 0.27	**0.86 ± 0.29**	**0.63 ± 0.11**	**0.01**
C15:0 (pentadecanoic)	0.19 ± 0.06	0.19 ± 0.06	0.17 ± 0.03	0.25
C16:0 (palmitic)	25.12 ± 2.49	**25.87 ± 2.26**	**22.69 ± 1.45**	**0.00**
C17:0 (margaric)	0.24 ± 0.08	0.24 ± 0.07	0.25 ± 0.11	0.88
C18:0 (stearic)	6.63 ± 0.80	6.69 ± 0.85	6.42 ± 0.63	0.32
C20:0 (arachidic)	0.06 ± 0.04	0.06 ± 0.04	0.06 ± 0.03	0.96
C21:0 (heneicosylic)	0.16 ± 0.06	0.16 ± 0.05	0.19 ± 0.09	0.07
C22:0 (behenic)	0.04 ± 0.01	0.03 ± 0.01	0.05 ± 0.00	0.22
C23:0 (tricosylic)	0.22 ± 0.13	0.24 ± 0.13	0.15 ± 0.10	0.06
C24:0 (lignoceric)	0.06 ± 0.02	**0.06 ± 0.02**	**0.07 ± 0.02**	**0.04**
MUFA				
C14:1 n-5 (myristoleic)	0.05 ± 0.03	0.05 ± 0.03	0.04 ± 0.02	0.32
C16:1 n-7 (palmitoleic)	3.14 ± 0.72	**3.27 ± 0.70**	**2.72 ± 0.60**	**0.02**
C17:1 n-10 (margarolein)	0.23 ± 0.09	0.23 ± 0.08	0.22 ± 0.10	0.63
C18:1 *tran*s n-9 (elaidic)	0.17 ± 0.12	0.18 ± 0.13	0.14 ± 0.08	0.34
C18:1 *cis* n-9 (oleic)	24.56 ± 2.04	**25.08 ± 1.93**	**22.86 ± 1.37**	**0.00**
cis-11 C20:1 n-9 (eicosenoic)	0.18 ± 0.07	0.19 ± 0.07	0.17 ± 0.03	0.35
cis-13 C22:1 n-9 (erucic)	0.08 ± 0.07	0.09 ± 0.07	0.08 ± 0.08	0.86
PUFA				
C18:2 n-9 (linolelaidic)	3.05 ± 0.36	3.08 ± 0.35	2.98 ± 0.41	0.40
C18:2 n-6 (LA; linoleic)	20.82 ± 2.65	**19.99 ± 2.38**	**23.52 ± 1.46**	**0.00**
C18:3 n-6 (GLA; γ-linolenic)	0.20 ± 0.12	0.19 ± 0.10	0.25 ± 0.15	0.09
C18:3 n-3 (ALA; α-linolenic)	0.54 ± 0.17	0.52 ± 0.16	0.61 ± 0.19	0.09
c9,t11-CLA (conjugated linoleic)	0.10 ± 0.04	0.11 ± 0.04	0.09 ± 0.03	0.31
C20:2 n-6 (eicosadienoic)	0.10 ± 0.08	0.10 ± 0.09	0.12 ± 0.06	0.42
C20:3 n-6 (dihomo-γ-linolenic)	1.10 ± 0.29	1.07 ± 0.28	1.20 ± 0.32	0.20
C20:3 n-3 (eicosatrienoic)	0.13 ± 0.16	0.13 ± 0.18	0.16 ± 0.07	0.62
C20:4 n-6 (AA; arachidonic)	3.82 ± 1.20	**3.36 ± 0.69**	**5.34 ± 1.24**	**0.00**
C20:5 n-3 (EPA; eicosapentaenoic)	0.37 ± 0.19	**0.33 ± 0.17**	**0.50 ± 0.19**	**0.01**
C22:2 n-6 (docosadienoic)	0.29 ± 0.17	0.31 ± 0.16	0.24 ± 0.18	0.23
C22:6 n-3 (DHA; docosahexaenoic)	0.77 ± 0.36	**0.66 ± 0.25**	**1.13 ± 0.44**	**0.00**
Total content				
∑ SFA	37.59 ± 3.12	**38.56 ± 2.81**	**34.44 ± 1.61**	**0.00**
∑ MUFA	28.36 ± 2.40	**29.03 ± 2.20**	**26.17 ± 1.63**	**0.00**
∑ PUFA	31.23 ± 3.77	**29.74 ± 2.80**	**36.09 ± 1.98**	**0.00**
∑ n-3	1.78 ± 0.67	**1.60 ± 0.56**	**2.36 ± 0.71**	**0.00**
∑ n-6	25.92 ± 3.56	**24.57 ± 2.77**	**30.31 ± 1.91**	**0.00**
n-6/n-3	16.21 ± 5.18	16.89 ± 5.21	14.01 ± 4.63	0.09

The values are given as the means ± standard deviations (SD). Bold data indicate significance difference. Mann–Whitney rank tests and *t*-tests were used where appropriate. *P* values were considered significant when *p* < 0.05.

We assessed the relationship between C-reactive protein (CRP) concentration and the fatty acid profile, and selected biochemical and anthropometric parameters in obese patients. Specifically, a positive correlation between CRP levels and the percentage of palmitic acid (*r* = 0.2965; *p* = 0.035) and IL-6 (*r* = 0.2902; *p* = 0.045) concentrations were found. However, only LA was an independent negative correlate of CRP (*r* =  − 0.3450; *p* = 0.013). The other parameters analyzed did not correlate statistically significantly with the CRP concentrations.

**Fig. 1:**
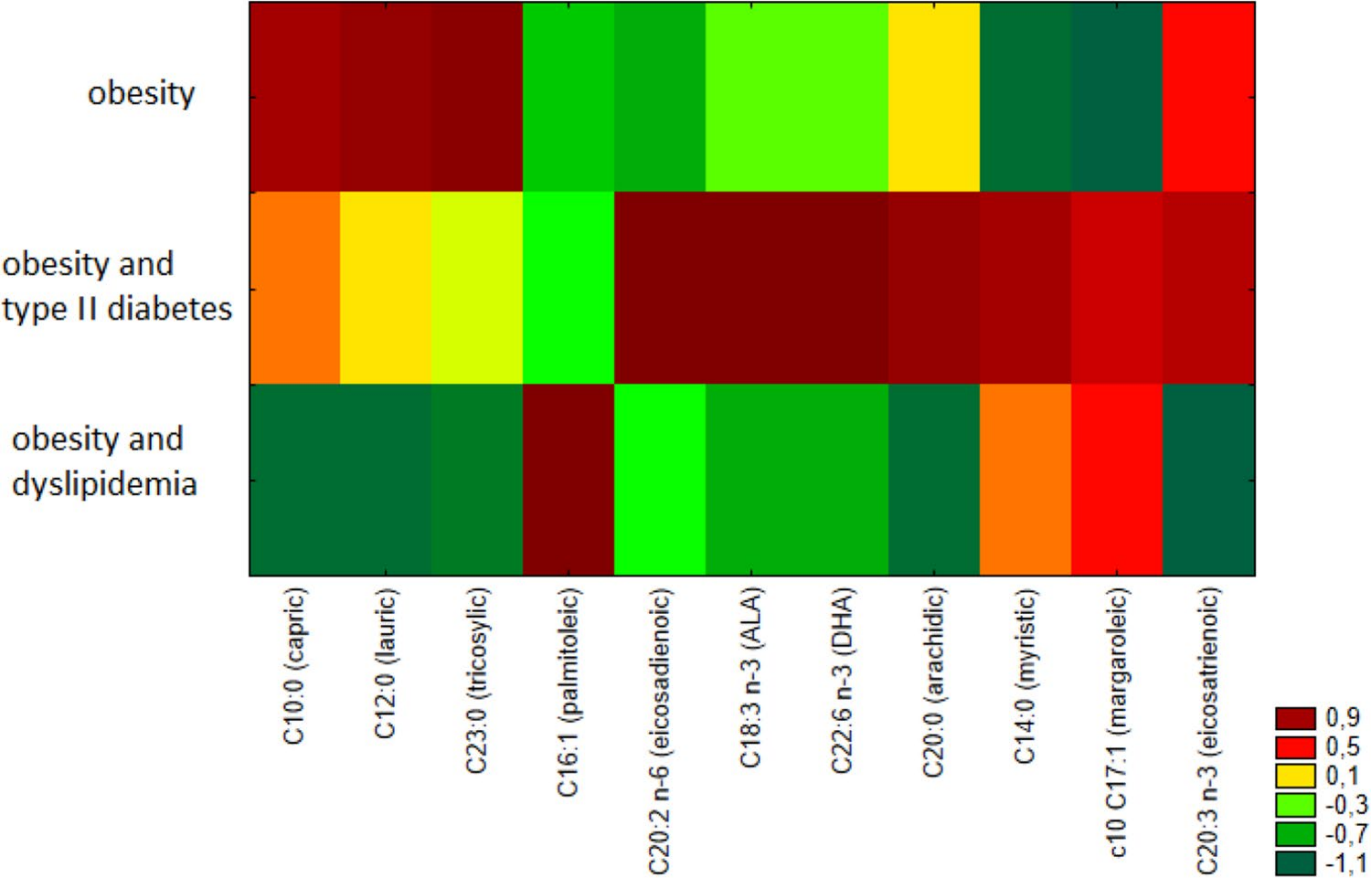
Heatmap of fatty acids percentage share differing significantly among three groups of obese patients (patients with obesity, with obesity and type II diabetes, with obesity and dyslipidemia)

We grouped participants into three categories: the first consisted of patients with obesity without diabetes/dyslipidemia (*n* = 27), the second patients with obesity and type II diabetes (without dyslipidemia, *n* = 6), and the third patients with obesity and dyslipidemia (without type II diabetes, *n* = 13). Patients with coexisting diabetes and dyslipidemia were excluded from the analyses. The heatmap visualization showed discrimination between analyzed groups of patients (Fig. 1).

Patients with extreme obesity and diabetes showed significantly higher percentages of the following fatty acids, C20:2 (eicosadienoic), C18:3 n-3 (ALA, α-linolenic), DHA (docosahexaenoic), C20:0 (arachidic), C14:0 (myristic), and C20:3 n-3 (eicosatrienoic), which distinguished them from others (Fig. 1). Likewise, they had significantly higher average total percentages of n-3 acids (∑n-3% = 2.47 ± 0.65) than obese non-diabetic patients (∑n-3% = 1.71 ± 0.63%; p = 0.01) and lower n-6/n-3 fatty acid ratio than obese non-diabetic patients (10.66 ± 2.75 vs. 16.78 ± 5.09; *p* < 0.01). The above relationships could be due to the fact that patients with type II diabetes more often implemented healthy lifestyle and diet principles, which, apart from pharmacotherapy, are the basis for treatment of type II diabetes, and this may have had a positive effect on the fatty acid profile in these patients. However, patients with diabetes were characterized by statistically significantly higher body weight, and higher IL-6, fasting glucose, glycated hemoglobin levels than patients without type II diabetes (data not shown).

The characteristics of fatty acid profiles in relation to the occurrence of dyslipidemia were also analyzed. Similarity analysis revealed that serum samples of patients with dyslipidemia were characterized by a lower percentage of such saturated acids as C10:0 (capric), C12:0 (lauric), C20:0 (arachidic), and C23:0 (tricosylic) and a higher percentage of such MUFA as C16:1 (palmitoleic) (Fig. 1). Such relationships may have resulted from the hypolipidemic treatment implemented in these patients [34].

In the next step, LDA was used to obtain appropriate classification rules for patients from above-mentioned groups based on examination of fatty acid share in their serum samples. Relevant discriminant functions were calculated in a stepwise progressive method. Percentage share of 30 fatty acids, which were detected in all examined serum samples, were included in the model. In the performed analysis, 13 variables have been included in the final model, and 6 of them (eicosadienoic, myristic, arachidic, c9,t11 CLA, heneicosylic, and pentadecanoic acids) were significant in the model. All of them made a comparable contribution to overall discrimination. Applied canonical analysis allowed to distinguish 2 statistically significant discriminant functions (DF). DF1 is the most significant function, as it explains 75.5% of discriminatory power, whereas DF2 explains 24.5% of discriminatory power (Table 3).

**Table 3:** Coefficients and average value of canonical variables included in the final model

Coefficients of canonical variables		
Variable (discriminatory power)	DF1 (75.5%)	DF2 (24.5%)
C20:2 n-6 (eicosadienoic)	− 0.613577	0.126347
C14:0 (myristic)	− 0.667717	0.420873
C20:0 (arachidic)	− 0.880123	-0.515471
C22:6 n-3 (DHA; docosahexaenoic)	− 0.568455	-0.129122
C12:0 (lauric)	− 0.141228	-0.461123
c9,t11-CLA (conjugated linoleic)	1.214006	0.029185
C21:0 (heneicosylic)	− 0.622736	0.089075
C15:0 (pentadecanoic)	− 0.645252	-0.211645
C23:0 (tricosylic)	0.314426	-0.647146
C18:2 n-6 (LA; linoleic)	− 0.662071	0.161652
C20:4 n-6 (AA; arachidonic)	0.955566	-0.725548
C24:0 (lignoceric)	− 0.725548	0.268595
C18:1 trans n-9 (elaidic)	0.408686	0.060113
Average value of canonical variables		
Dyslipidemia	0.75753	1.467863
Diabetes	− 4.34991	0.099946
Obesity	0.60191	− 0.728959

**Fig. 2:**
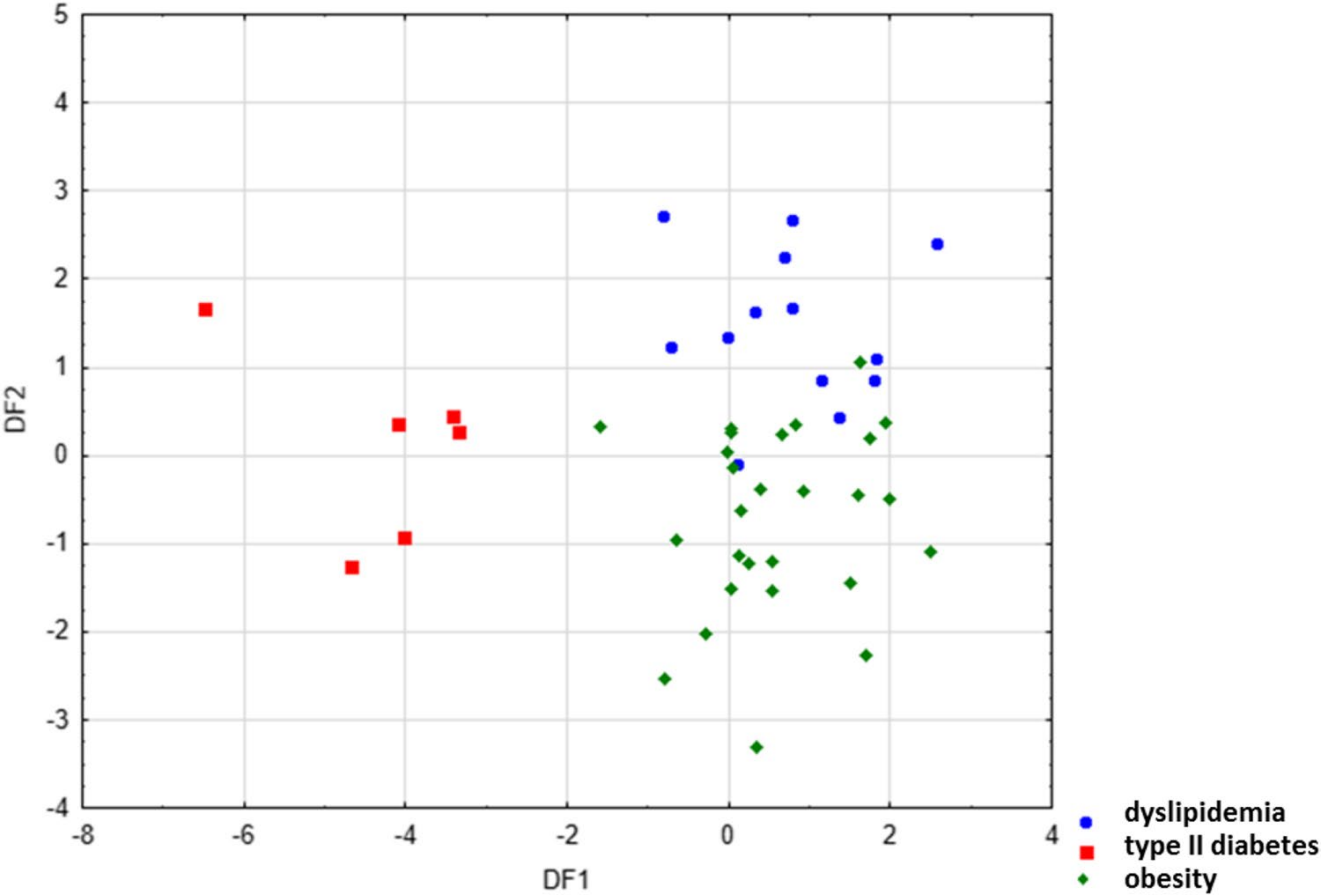
Scatter plot of canonical values for functions DF1 and DF2

Analysis of canonical mean variables indicated that DF1 had the greatest impact on the distinction of serum samples obtained from patients with obesity and type II diabetes, whereas DF2 seemed to distinguish patients with obesity and dyslipidemia from obese patients, without coexisting diabetes or dyslipidemia. Graph analysis confirms the suggestion provided by the values of average canonic variables (Fig. 2).

The calculated classification matrix indicated that average classification efficiency based on the calculated functions was 93.5%. For individual groups, these coefficients were as follows: 100% for obese patients with diabetes, 96% for obese patients without coexisting diabetes or dyslipidemia, and 85% for obese patients with dyslipidemia. Thus, the applied LDA allowed to observe significant differences among all three distinguished groups of patients which indicated that the analysis of fatty acid profile in serum samples allowed for the distinction of the origin of the sample with high probability. The fatty acid profile of serum samples elaborated with various chemometric methods can be used to some extent to differentiate health status of obese patients.

A comparison of serum fatty acid profiles in patients at baseline and at a follow-up visit (*n* = 23), 1 year after laparoscopic sleeve gastrectomy (LSG), is presented in Table 4. The mean BMI value of the subjects at 12 months post‐surgery was 31.73 ± 4.76 kg/m^2^. Mean weight loss was 34.8 kg (− 46.6%) during the first year after surgery. The observed weight reduction had an effect on the fatty acid profile. Compared to baseline, patients after bariatric surgery had significantly lower percentage shares of such SFA as C8:0 (caprylic), C10:0 (capric), C12:0 (lauric), C14:0 (myristic), C16:0 (palmitic), and C23:0 (tricosanic); and of MUFA such as C14:1 (myristoleic), C16:1 (palmitoleic), and C18:1 trans n-9 (elaidic); as well as a lower total percentage of serum SFA. Significant weight reduction achieved as a result of bariatric surgery also resulted in a statistically significantly lower total percentage of MUFA. Patients after the bariatric surgery were characterized by a significantly higher total percentage of PUFA. Specifically, they had higher percentages of linoleic, arachidonic, and DHA (docosahexaenoic) compared to patients at baseline (Table 4).

**Table 4:** Changes in fatty acid composition in serum after weight loss (WL) achieved by laparoscopic sleeve gastrectomy (LSG)

Fatty acids (%)	Baseline	After WL	Change	*p value*
SFA				
C6:0 (caproic)	n.d	n.d	-	-
C8:0 (caprylic)	1.47 ± 0.57	1.13 ± 0.80	-0.34	0.04
C10:0 (capric)	1.67 ± 0.48	1.21 ± 0.69	-0.46	0.01
C11:0 (undecylic)	n.d	n.d	-	-
C12:0 (lauric)	**0.92 ± 0.26**	**0.64 ± 0.33**	**-0.28**	**0.00**
C13:0 (tridecylic)	0.13 ± 0.19	0.12 ± 0.13	-0.01	0.82
C14:0 (myristic)	**0.80 ± 0.34**	**0.63 ± 0.32**	**-0.18**	**0.03**
C15:0 (pentadecanoic)	0.18 ± 0.05	0.19 ± 0.06	0.00	0.69
C16:0 (palmitic)	**25.81 ± 2.74**	**23.68 ± 2.41**	**-2.13**	**0.00**
C17:0 (margaric)	0.25 ± 0.09	0.25 ± 0.07	0.00	0.81
C18:0 (stearic)	6.62 ± 0.84	6.51 ± 0.61	-0.11	0.47
C20:0 (arachidic)	0.05 ± 0.04	0.06 ± 0.09	0.02	0.47
C21:0 (heneicosylic)	0.17 ± 0.07	0.15 ± 0.04	-0.02	0.27
C22:0 (behenic)	n.d	n.d	-	-
C23:0 (tricosylic)	**0.24 ± 0.10**	**0.14 ± 0.09**	**-0.10**	**0.00**
C24:0 (lignoceric)	0.05 ± 0.01	0.04 ± 0.01	-0.01	0.30
MUFA				
C14:1 n-5 (myristoleic)	**0.04 ± 0.02**	**0.03 ± 0.02**	**-0.02**	**0.00**
C15:1 n-10 (pentadecenoic)	n.d	n.d	-	-
C16:1 n-7 (palmitoleic)	**3.16 ± 0.74**	**2.24 ± 0.80**	**-0.91**	**0.00**
C17:1 n-10 (margarolein)	0.24 ± 0.10	0.26 ± 0.15	0.02	0.58
C18:1 *tran*s n-9 (elaidic)	**0.20 ± 0.15**	**0.10 ± 0.04**	**-0.10**	**0.03**
C18:1 *cis* n-9 (oleic)	24.52 ± 1.97	24.14 ± 2.60	-0.38	0.39
cis-11 C20:1 n-9 (eicosenoic)	**0.18 ± 0.05**	**0.22 ± 0.09**	**0.04**	**0.01**
cis-13 C22:1 n-9 (erucic)	0.02 ± 0.00	n.d	-	-
C24:1 n-9 (nervonic)	n.d	n.d	-	-
PUFA				
C18:2 n-9 (linolelaidic)	3.07 ± 0.32	2.93 ± 0.37	-0.14	0.11
C18:2 n-6 (LA; linoleic)	**20.63 ± 3.32**	**24.37 ± 4.26**	**3.74**	**0.00**
C18:3 n-6 (GLA; γ-linolenic)	0.17 ± 0.13	0.16 ± 0.09	-0.01	0.61
C18:3 n-3 (ALA; α-linolenic)	0.53 ± 0.19	0.51 ± 0.33	-0.02	0.78
c9,t11-CLA (conjugated linoleic)	0.09 ± 0.03	0.10 ± 0.06	0.01	0.54
C20:2 n-6 (eicosadienoic)	0.09 ± 0.05	0.10 ± 0.06	0.01	0.41
C20:3 n-6 (dihomo-γ-linolenic)	0.96 ± 0.24	0.95 ± 0.24	-0.01	0.79
C20:3 n-3 (eicosatrienoic)	0.25 ± 0.35	0.16 ± 0.14	-0.08	0.62
C20:4 n-6 (AA; arachidonic)	**3.47 ± 1.14**	**4.68 ± 1.41**	**1.21**	**0.00**
C20:5 n-3 (EPA; eicosapentaenoic)	0.34 ± 0.20	0.34 ± 0.15	0.01	0.82
C22:2 n-6 (docosadienoic acid)	0.34 ± 0.18	0.22 ± 0.17	-0.12	0.11
C22:6 n-3 (DHA; docosahexaenoic)	**0.71 ± 0.34**	**0.90 ± 0.41**	**0.19**	**0.00**
Total content				
∑ SFA	**38.20 ± 3.11**	**34.61 ± 2.93**	**- 3.60**	**0.00**
∑ MUFA	**28.35 ± 2.35**	**26.97 ± 3.09**	**- 1.39**	**0.01**
∑ PUFA	**30.45 ± 4.23**	**35.27 ± 5.57**	**4.82**	**0.00**
∑ n-3	1.69 ± 0.68	1.80 ± 0.77	-0.11	0.39
∑ n-6	**25.19 ± 4.20**	**30.18 ± 5.45**	**4.99**	**0.00**
n-6/n-3	16.91 ± 6.01	18.87 ± 6.27	-1.95	0.09

Data expressed as mean ± SD; *p* values were considered significant when *p* < 0.05 (in bold). Values at baseline were compared with post‐surgery values using paired *t*-test

## Discussion

Our study showed that in the patients with clinically severe obesity before bariatric surgery, palmitic acid (C16:0) and oleic acid (C18:1 cis n-9) predominated among total plasma saturated fatty acids, comprising ≈ 25%, followed by linoleic acid (LA, C18:2 n-6, ≈ 21%) and stearic acid (C18:0, ≈ 7%). Our observations were similar to those of other authors. Mayneris-Perxachs et al. [35] found similar values in stearic acid (≈ 7%) and oleic acid (≈ 26%), but found lower values in palmitic acid (≈ 22%), and higher values in linoleic acid (≈ 28%) in a Mediterranean population with metabolic syndrome. In particular, we examined the concentration of circulating PUFA, which have two or more double bonds in the hydrocarbon chain, because the hallmarks for most metabolic pathologies are an increased content of SFA and a lower content of PUFA. Obese individuals in the highest quartile (Q3 quartile) of serum PUFA (% PUFA > 32.84%) had a significantly lower percentage of SFA, especially C14:0 (myristic), C16:0 (palmitic), and C24:0 (lignoceric), and lower percentages of selected MUFA; i.e., C16:1 n-7 (palmitoleic) and C18:1 cis n-9 (oleic). Similarly, previous studies showed that people who followed a diet rich in PUFA had lower serum content of palmitic [25, 36], palmitoleic, and oleic [25] acids than controls. This indicates that the measurement of fatty acid composition in serum is a highly objective method which can be comparable with intake of dietary fat. It should also be emphasized that a low content of C16:0 (palmitic acid) is critical because a relationship has been demonstrated between an elevated level thereof and the development of type II diabetes, cardiovascular disease, and cancer [37]. Palmitic acid stimulates pro-inflammatory mechanisms through both Toll-like receptor 4 (TLR4)-mediated inflammatory signaling [38] and reactive oxygen species (ROS) in a TLR-independent manner [39]; induces central leptin resistance; and impairs hepatic glucose and lipid metabolism [40]. The lower level of palmitoleic acid observed here was probably related to decreased palmitic acid intake, resulting in decreased endogenous desaturation of palmitic acid to palmitoleic acid. Moreover, our findings provide evidence for a positive correlation between serum C-reactive protein (CRP) concentrations and palmitic acid (C16:0) and IL-6 levels. This is in line with the fact that they are two functionally linked biomarkers and that hepatic synthesis of CRP is regulated by IL-6 [41, 42]. The functional framework between IL-6 and CRP [42] may be strongly influenced by the increased production of IL-6 by human adipose tissue in cases of obesity. Moreover, CRP has been shown to be expressed in adipocytes in response to pro-inflammatory mediators, representing yet another link between obesity and chronic inflammation [43]. On the other hand, patients with higher serum total PUFFA (> Q3 quartile) had a higher percentage of linoleic acid (LA), arachidonic acid (AA), EPA (eicosapentaenoic acid), and DHA (docosahexaenoic acid) compared to patients with lower PUFA levels (% PUFA ≤ Q3). The higher serum total n-3 fatty acid content in this group was accompanied by a higher proportion of total n-6 fatty acid in serum. Similar results were obtained by Summers et al., where individuals on a diet rich in PUFA had increased LA (linoleic acid) concentrations [25]. Several lines of evidence have indicated that, as with the n-3 fatty acids, the n-6 reduce risk for coronary heart disease (CHD) [44, 45]. In our study, the main n-6 PUFA, LA, was associated with lower CRP. When KIHD study’s participants were divided into four groups based on their serum LA (linoleic acid) levels, the probability for an elevated CRP was 53% lower in the highest quarter compared to the lowest one. Indeed, no evidence is available from randomized, controlled intervention studies to show that high intake of LA increases the concentration of inflammatory markers, including C-reactive protein, fibrinogen, plasminogen activator inhibitor type 1, cytokines, soluble vascular adhesion molecules, or tumor necrosis factor-α [46]. Moreover, in a study by Steffen et al., which involved 2848 adults from various ethnic groups, it was found that people with higher percentages of LA (linoleic acid) had statistically lower concentrations of CRP. In addition, plasma LA levels appear especially important among obese individuals in reducing the likelihood of high levels of sICAM-1 [47]. Also, Fernandez-Real et al. showed that serum CRP levels in individuals with obesity was negatively correlated with the percentage of LA (linoleic acid) (*p* = 0.03) [24].

In this study, patients with higher total PUFA percent had higher HDL-cholesterol levels and lower triglyceride concentrations, even though they had higher BMI, and this is consistent with the previous findings of Virtanen et al. [45]. Similarly, Hlavaty et al. [48] showed that moderately obese women assigned to a low calorie diet including yogurt enriched with n-3 PUFA showed a significant increase in HDL-cholesterol concentration and a decrease in triglyceride levels after 21 days. It has been shown that fatty acid component of structured triacylglycerols influences its digestion and absorption; moreover, PUFA are regulators of the expression of genes encoding proteins involved in energy metabolism and reduce triglyceride levels in plasma [49–51]. In addition, in our study, we can exclude the influence of cholesteryl ester-transfer protein (CETP) polymorphism on presented results. However, the lack of association between CETP Taq1B-variant and total PUFFA level should be treated as preliminary, because of small group of patients with B2B2 genotype. In general, cholesteryl ester-transfer protein (CETP) transfers cholesteryl esters from HDL to apolipoprotein B containing lipoproteins in exchange for triglycerides. Previous studies showed that the B2 allele in the CETP gene was associated with decreased CETP activity and increased HDL-C-cholesterol [52].

The serum fatty acid profile has previously been studied in patients with type II diabetes [53–56]. Some studies suggest that SFA can be associated with diabetes risk. In the present study, obese patients with type II diabetes were characterized by higher percentages of the following SFA: C14:0 (myristic), C20:0 (arachidic), and C21:0 (heneicosylic). However, we are not in accord with regard to the differences seen previously in the other SFA; e.g., C16:0 (palmitic) [53, 55, 56] and C18:0 (stearic) [55, 56], between patients with and without type II diabetes. Similar to our data, a study conducted in England by Patel et al. [53] found increases in the percentages of myristic acid (14:0) and α-linolenic acid (ALA, 18:3n-3) in patients with type II diabetes. In contrast, we found also DHA and total n-3 fatty acids to be increased in the patients with obesity and diabetes. In a study carried out in Australia, Hodge et al. [55] found increases in most fatty acids in patients with type II diabetes, including EPA and DHA. Thus, serum SFA level may be an important underlying cause of various obesity-associated conditions. The occurrence of obesity is usually accompanied by lipid metabolism disorders. We analyzed the fatty acid profile in the serum of obese patients with and without dyslipidemia. We noticed that patients with lipid metabolism disorders had significantly lower percentages of some SFA in the serum, such as C10:0 (capric), C12:0 (lauric), and C20:0 (arachidic). Previous studies have shown an increased level of total MUFA as the effect of hyperlipidemia [57], which is in agreement with our results for higher percentage of palmitoleic acid (C16:1). Such interaction may have resulted from the hypolipidemic treatment implemented in these patients [34]. Coupled with various chemometric methods, linear discriminant analysis (LDA) may be served as method to differentiate health status of patients with obesity. LDA allowed to distinguish with high probability the origin of the serum sample obtained from three distinguished groups of patient: obese patients, obese patients with type II diabetes, and obese patients with dyslipidemia. Previously, LDA has been proposed as a useful tool in different disorders diagnosis [58–60]. Further studies are necessary in order to conclude a final association between the FA profile and the prevalence of any obesity-related complications. Evaluating these differences may be crucial in the approach to the candidate for bariatric surgery.

In order to maintain a good state of health, it is important to keep the dietary ratio of n-6 acids to n-3 low. High ratio may contribute to the development of cardiovascular diseases, cancers, inflammation, and autoimmune diseases [61]. In the present study, the average ratio of n-6/n-3 fatty acids was 16.21 ± 5.18 in obese patients. Gupta et al. observed a relationship between the high ratio of n-6/n-3 fatty acids and the occurrence of dyslipidemia in the population of Delhi [62]. In this study, it was observed that obese patients with dyslipidemia were characterized by a higher ratio of n-6/n-3 fatty acids than patients with diabetes type II. It must be said that diabetics more effectively control their diets, because they are instructed not to eat excessive and fatty meals. That this instruction was quite well respected can be seen from the lower ratio of n-6/n-3.

The results of many studies confirm the improvement of lipid parameters after weight reduction, e.g., due to surgical gastrectomy [63–67]. We also noted that patients after the 12-month period following LSG had more favorable percentages of some fatty acids. The examined patients were characterized by a statistically significant lower total percentage of saturated fatty acids (SFA), as well as lower percentages of individual SFA, such as C8:0 (caprylic), C10:0 (capric), C12:0 (lauric), C14:0 ( myristic), C16:0 (palmitic), and C23:0 (tricosanic). Walle et al. also noted a statistically significant reduction in the total concentration of saturated fatty acids, especially C16:0 (palmitic) acid, 1 year after bariatric surgery. Such changes induced by obesity surgery are especially beneficial because palmitic acid in adipose tissue is negatively associated with insulin sensitivity. Similar to the study conducted by Walle et al. [68], who examined 122 people after surgical gastrectomy, we likewise observed that patients after surgical gastrectomy had higher concentrations of C20:1 (eicosenic), AA (arachidonic), DHA (docosahexaenoic), and LA (linoleic) acids, and higher total PUFA concentrations, than patients before surgery. This observation in our patients after bariatric surgery is attributed to the higher amounts of LA, which is a precursor of arachidonic acid. In general, AA has been characterized as an inflammation inducer due to its conversion into pro-inflammatory prostaglandins and leukotrienes. However, AA metabolism is a rigidly regulated process [69], and moderate elevations in body’s AA concentrations in serum may have little to no net effect on the induction of pathophysiological processes. Alternatively, AA is a potent bioactive mediator that also regulates physiological processes, such as the production of anti-inflammatory lipoxin A4 [70] and the inhibition of the transcription factor nuclear factor-κB [71], which is associated with production and release of a number of cytokines, including IL-6. Moreover, after weight loss achieved by laparoscopic sleeve gastrectomy, we recognize decrease in major monounsaturated trans-fatty acid, elaidic acid, which is a reliable biomarker of highly processed foods [72], and may induce weight gain [73]. Thus may be of importance in maintaining weight loss.

Our study has several strengths: it included both obese patients with hyperlipidemia and with diabetes type II and had a good follow-up rate. However, further study is warranted to confirm our observation in changes in serum fatty acid levels during the first year after bariatric surgery, which suggests differences in fatty acid metabolism, and may also have implications in dietary fatty acid intake recommendations for obese individuals after bariatric surgery.
